# Medical Institute of Louisville—Professor of Surgery

**Published:** 1840

**Authors:** 


### MEDICAL INSTITUTE OF LOUISVILLE.

New Charter.—The last session of our Legislature gave to the Medical Institute of Louisville, a new charter, in which the power to confer the degree of *Doctor of Medicine* (previously exercised under a sound construction of the law of 1835) was given in express terms.

Professor of Surgery.—Dr. Flint having resigned the Chair of *Surgery* in the Institute, Samuel D. Gross, M. D., late Professor in the Cincinnati Medical College, has been called to the vacancy. Dr. Gross possesses eminent skill as a practitioner, and is extensively and favorably known as a popular and able writer and teacher. His appointment will increase the claims of the Institute to the confidence and support of the profession.

Dr. Gross commenced his career as a public teacher in 1833, when he was invited by Dr. Eberle from Pennsylvania to take the office of *Demonstrator of Anatomy*, in the Medical College of Ohio. His accurate knowledge of the science, his skill in dissection, and his facility in lecturing, gave him immediate reputation; and, in 1835, when the Medical Department of the Cincinnati College was instituted he was unanimously appointed professor of *General and Pathological Anatomy*, *Physiology and Medical Jurisprudence*, which chair he filled, with distinguished success, till the department was suspended in 1839.

Dr. Gross entered on the duties of the profession, as a surgeon and physician in 1828, and has for some time been in extensive practice; but in the midst of professional duties, in the comparatively short period of twelve years, he has been enabled to prepare for publication the following works; many of which have been widely circulated among the profession.

TRANSLATIONS.

1. *A Manual of General and Pathological Anatomy.*—From the French of A. L. J. Bayle & H. Hollard. Philadelphia: 1828.

2. *A Manual of Obstetrics.*—From the French of Julius Hatin. Philadelphia: 1828.

3. *A Treatise on Contagious Typhus.*—From the German of J. Val. De Hildenbrand. New York: 1829.

4. *Elements of Operative Surgery.*—From the French of A. Tavernier. Philadelphia: 1829.

ORIGINAL WORKS.

1. *The Anatomy, Physiology and Diseases of the Bones and Joints.* Philadelphia: 1830.

2. *Elements of Pathological Anatomy*, in 2 vols. Boston, 1839.

The last work, although not published till near the end of the year 1839, has already been made a text book in four Medical schools. It may be said to embrace the *Institutes of Surgery.*

In addition to these different works, Prof. Gross is the author of several papers in the journals of the day, one of which is an original inquiry into the signs and morbid appearances, in cases of death from *manual strangulation.* Y.

